# Protein Fractionation of Green Leaves as an Underutilized Food Source—Protein Yield and the Effect of Process Parameters

**DOI:** 10.3390/foods10112533

**Published:** 2021-10-21

**Authors:** Anna-Lovisa Nynäs, William R. Newson, Eva Johansson

**Affiliations:** Department of Plant Breeding, Swedish University of Agricultural Sciences, P.O. Box 190, SE-23422 Lomma, Sweden; bill.newson@slu.se (W.R.N.); eva.johansson@slu.se (E.J.)

**Keywords:** leaf protein extraction, RuBisCO, green biorefinery, leaf protein concentrate, white protein precipitation, thermal protein precipitation

## Abstract

Green biomass has potential as a sustainable protein source for human consumption, due to its abundance and favorable properties of its main protein, RuBisCO. Here, protein fractionation outcomes of green leafy biomass from nine crops were evaluated using a standard protocol with three major steps: juicing, thermal precipitation, and acid precipitation. Successful protein fractionation, with a freeze-dried, resolubilized white protein isolate containing RuBisCO as the final fraction, was achieved for seven of the crops, although the amount and quality of the resulting fractions differed considerably between crops. Biomass structure was negatively correlated with successful fractionation of proteins from biomass to green juice. The proteins in carrot and cabbage leaves were strongly associated with particles in the green juice, resulting in unsuccessful fractionation. Differences in thermal stability were correlated with relatedness of the biomass types, e.g., *Beta vulgaris* varieties showed similar performance in thermal precipitation. The optimal pH values identified for acid precipitation of soluble leaf proteins were lower than the theoretical value for RuBisCO for all biomass types, but with clear differences between biomass types. These findings reveal the challenges in using one standard fractionation protocol for production of food proteins from all types of green biomass and indicate that a general fractionation procedure where parameters are easily adjusted based on biomass type should instead be developed.

## 1. Introduction

In Europe and beyond, consumer preferences are shifting to increased consumption of plant-based instead of animal-based protein [1,2]. This shift has resulted in a multitude of novel products appearing on supermarket shelves. However, these novel food products, like protein-based animal feed products currently in use, are largely based on soy protein [3], the majority of which is grown in America and Asia [4]. Thus, replacement of soy with locally produced plant protein would contribute to food independence for the country of production, while a reduction in transportation could lead to increased sustainability [5]. Valorization of green leaves by protein extraction has been a concept since the 1940s [6], but has gained increasing attention in recent decades as an additional plant protein source for food and feed [7,8]. Green leaves are a major source of biomass worldwide and also one of the largest side-streams from modern agricultural and horticultural production. For example, only 20–50% of total plant biomass of broccoli is currently harvested and used for food [9], and similar percentages can be expected for crops such as carrot, beetroot, sugarbeet, and cabbage. Increased and diversified use of the residual side-streams would increase sustainability and profitability in crop production.

Fresh green leaves consist of 1.6–8.2% protein, with large variation between species [8]. The major protein in all green leaves is the enzyme ribulose-1,5-bisphosphate carboxylase/oxygenase (RuBisCO), considered the most abundant protein in the world [10]. RuBisCO catalyzes uptake of CO_2_ during photosynthesis and is therefore present in high amounts in all photosynthetic organisms. RuBisCO is an interesting target protein as a source for novel protein-rich foods, as it has a highly desirable amino acid composition for human consumption and functional properties resembling those of soy and whey protein [11,12,13].

Protein fractionation can be utilized to create value-added products from leaf proteins, resulting in (i) a green protein fraction, mainly consisting of membrane-bound proteins and chlorophyll-related proteins, and (ii) a “non-green” protein fraction of water-soluble proteins, mainly RuBisCO [14]. This fraction is commonly referred to as “white protein”, a convention followed in this paper. The white protein fraction has been shown to be beneficial for humans, with high levels of essential amino acids [13,15,16]. It has also been shown to have functional properties useful in food applications, e.g., foaming, emulsification, and gelation [11,12,13,16,17,18].

**Figure 1:**
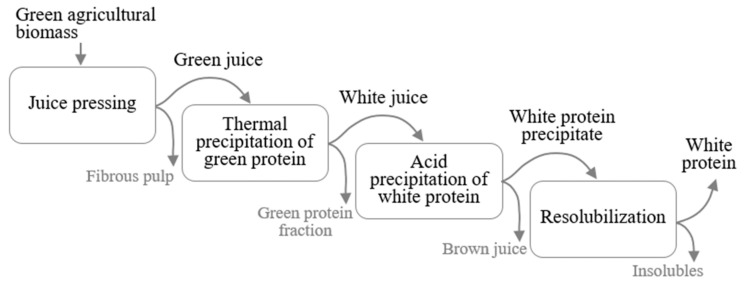
Main steps in a general process for producing white protein isolate from green agricultural biomass.

Most published studies on protein fractionation from green biomass for food applications have applied a relatively gentle extraction process, to maintain the desirable functional properties of the proteins [18]. This process generally consists of three main steps (Figure 1): (i) pressing liquid from fresh or frozen green leaves to obtain a green juice (GJ); (ii) separating the green and white protein fractions by exploiting differences in thermal sensitivity of the proteins, to create white juice (WJ) and a green protein fraction [11,13,18,19,20,21]; and (iii) concentrating the white protein fraction in the WJ further, by heating at 80 °C [19], acid precipitation at pH between 3.5 and 4.5 [13,16,22,23,24], chromatography [20], or filtration [11,17].

Industrial-scale protein fractionation of green leaves for food would require a year-round process where a range of different biomass types are used in a similar methodological system. In a Nordic context, biomass availability will clearly vary between seasons and a biorefinery would benefit from having multiple raw materials available. However, previous studies have mainly focused on green biomass of three crops (lucerne, sugarbeet, and spinach) for protein fractionation [11,13,15,16,17,18,21,23,24,25,26,27,28,29,30,31,32], with only a few studies focusing on other biomass sources (e.g., beetroot, broccoli, cauliflower, or cabbage leaves [33]). Thus, there is limited information available on how different biomass sources perform as raw material in protein extraction and how the extraction process may need to be adapted for individual sources.

The aim of the present study was to evaluate the role of green biomass source in protein fractionation and to characterize the background and underlying reasons for any differences between biomass types. In characterization, the emphasis was on protein precipitation temperatures suitable pH for white protein precipitation and on the flow of water, dry matter, and nitrogen through the extraction process. An overarching goal was to establish a basis for industrial use of green agricultural waste/side-streams for protein valorization into food ingredients, additives, and products. The starting hypothesis was that any green leafy biomass can be used as raw material in a general protein extraction process.

## 2. Materials and Methods

### 2.1. Collection of Biomass

To evaluate similarities and differences between a wide array of green biomass sources of possible use in a Nordic set-up for protein fractionation, nine different crops were selected (Table 1). Fresh leafy harvest residues from broccoli, cabbage, kale, carrot, beetroot, and sugarbeet were collected from fields in southern Sweden at the time of harvest. The mangold residues were over-mature and not suitable as a food ingredient, mainly due to cosmetic reasons, but did have a value as a green biomass. Lucerne (first cut) was collected from a field on campus in Alnarp, Sweden. Fresh baby spinach was included in the study as a model crop, as it has previously been shown to perform well in a protein extraction process, it was purchased from a supermarket. After collection, all biomass was rinsed with tapwater, frozen, and stored at −20 °C until processing. Samples for dry matter and nitrogen determination were collected prior to freezing, the details are described in Section 2.4. Further processing was done following the procedure described in Section 2.2, based on process parameters developed in Section 2.3.

### 2.2. Protein Fractionation from Green Leaves

**Figure 2:**
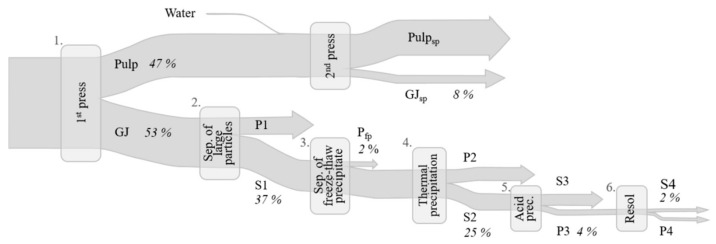
Sankey chart of the full protein extraction process, including all additional steps, with values for mangold as an example. Percentages shown indicate nitrogen flow through the process steps 1–6 (% of initial biomass nitrogen). GJ: green juice, S: supernatant, P: pellet, sp: second press, fp: freeze precipitate.

To enable direct comparisons of different green leaf sources during protein fractionation, the same protein fractionation procedure was applied for all crops. The methodology selected was mainly based on literature data [18,24,28,31], although with some modifications. In principle, the fractionation procedure comprised three steps; screw pressing, thermal precipitation, and acid precipitation (see Section 2.2.1, Section 2.2.2 and Section 2.2.3). To investigate the background and reasons for differences in biomass behavior during protein fractionation, additional steps were included (see Section 2.3). A Sankey chart of the full process is shown in Figure 2.

#### 2.2.1. Juice Pressing

Frozen leaves of the selected crops were thawed and divided into three 300 g portions, representing process triplicates. Thawed, but still cold, leaves were juiced in a twin-screw press (Angelia 5500, Angel Co., Ltd., Busan Korea), and the green juice (GJ) obtained was centrifuged (11,800 RCF, 10 min, 4 °C), producing a particle-free supernatant (S1) containing water-soluble proteins, and a pellet with particles (P1). The extruded fibrous pulp was mixed with MilliQ water corresponding to 50% (or 100% for carrot) of its mass after sampling and re-fed to the juicer. As in the first juicing, the second press resulted in a green juice (GJ_sp_), which was collected and centrifuged into an S1_sp_ and P1_sp_ fraction. Portions of all samples were freeze-dried for dry matter determination and analysis of nitrogen content. The additional S1 juices were stored at −20 °C until further processing.

#### 2.2.2. Thermal Precipitation

Subsamples of thawed S1 juice (40 mL) from each crop and process replicate were transferred to triplicate 50 mL conical tubes and centrifuged (3200 RCF, 10 min, 4 °C) to remove particles resulting from protein precipitation during freezing (P_fp_). The supernatant in each tube was transferred to a new tube and thermally treated in a water bath at 55 °C for 25 min. The thermally treated samples were immediately cooled on ice and centrifuged (3200 RCF, 10 min, 4 °C), resulting in a supernatant (S2) containing the white protein fraction, and a pellet (P2) containing the green protein fraction. The supernatants were frozen and stored at −20 °C until further processing. Aliquots of the S2 supernatant and the pellets (P_fp_ and P2) were freeze-dried and analyzed for dry matter and nitrogen content, while the remaining S2 supernatant was retained for further processing.

#### 2.2.3. Acid Precipitation and Resolubilization of the White Protein Fraction

Part of the S2 fraction of each crop and their process replicates were pH-adjusted to 4.5 by drop-wise addition of 1 M HCl, and the solution was divided into (A) a sample with total volume 1.5 mL for monitoring precipitation yield, and (B) a sample with volume 8.0 mL intended for resolubilization of the precipitated white protein (see Figure 2). The precipitated particles in both sample sets were separated by centrifugation (1150 RCF, 10 min, 4 °C), and the supernatant (S3) was separated from the pellet (P3). The S3 and P3 from sample A were weighed and frozen. The P3 from sample B was dispersed in 1 mL MilliQ water and the pH of the solution was adjusted to 7 through drop-wise addition of 0.1 M NaOH. Thereafter the sample was stirred at room temperature for 30 min, followed by gentle centrifugation (260 RCF, 5 min, 4 °C) to remove insoluble particles. The supernatant (S4) was poured off and the pellet (P4) was washed by addition of 1 mL MilliQ water, mixing, and another centrifugation step. The supernatant was then pooled with the first S4. To assess unintended protein precipitation due to freezing, a 1.5 mL sample of S2 from each crop and process replicate was treated following the same procedure as described for sample A, but without pH adjustment. The supernatant and pellet from this test are referred to as S_fp2_ and P_fp2_. All samples produced (S3 and P3 from sample A, S_fp2_, P_fp2_, S4, and P4) were freeze-dried and the dry matter and nitrogen content were determined.

### 2.3. Thermal and Acid Precipitation Tests to Determine Differences between Biomass Sources

#### 2.3.1. Thermal Precipitation

The S1 fraction from the different crops and their processing replicates were thawed on ice and 100 µL aliquots were transferred to 300 µL micro-Eppendorf tubes. For each crop and processing replicate, three tubes per temperature were placed in a water bath at 40, 45, 50, 55, 60, 65, 70, or 80 °C for 10 min, and placed on ice immediately afterwards. A reference sample corresponding to 0 °C was left on ice. The samples were centrifuged (1900 RCF, 10 min, 4 °C) to separate the precipitated protein from the soluble proteins. The protein concentration in the supernatant of each sample was analyzed in triplicate using a bicinchoninic acid protein assay kit (Pierce BCA protein assay, Thermo Scientific, USA) in 96-well format according to the manufacturer’s instructions, using a Multiskan GO spectrophotometer (ThermoFisher Scientific, Vantaa, Finland).

#### 2.3.2. Acid Precipitation

Part of the S2 fraction was filtered through a 0.45 µm syringe filter to remove particles. The filtrate was diluted in MilliQ water to reach a protein concentration of around 1 mg/mL (W_prot_/V), transferred to a disposable capillary zeta cell (Malvern Instruments, Malvern, UK), and analyzed with a Zetasizer nano ZS (Malvern Instruments, Malvern, UK) coupled with an autotitration unit (MPT-2, Malvern). The pH of the solution was changed in steps of 0.5 units using 0.1 and 0.01 M HCl, and particle size and their zeta potential (ZP) were measured at each step. Duplicate measurements were made for two process replicates, and at each pH value triplicate particle size and ZP measurements were averaged.

#### 2.3.3. SDS-PAGE Analysis

Sodium dodecyl sulfate–polyacrylamide gel electrophoresis (SDS-PAGE) analysis was performed using an analysis kit with pre-cast gradient mini-gels (Invitrogen Novex Bolt, 4–12%, Bis-Tris Plus, Thermo Fisher Scientific, Waltham, MA, USA), and a protein ladder (Invitrogen SeeBlue^®^). A voltage of 145 V was applied during 35 min for separation. The protein bands were stained for 15 min (GelCode^TM^ Blue Safe protein stain, Thermo Scientific, USA), and the gels were washed overnight. For SDS-PAGE analysis of freeze-dried protein concentrates, the material was dissolved in MilliQ water to reach a concentration of 4 mg dry material/mL and the samples were mixed at room temperature for 10 min before analysis. In SDS-PAGE analysis, the RuBisCO subunits are found at ~55 kDa and ~14 kDa [10].

### 2.4. Dry Matter and Nitrogen Determinations

Fresh biomass samples of 3 or 5 g were oven-dried in duplicate or triplicate at 110 or 130 °C. All other samples were freeze-dried. All samples were weighed before and after drying, and the dry matter content was calculated. Nitrogen (N) determination was carried out on dried samples through applying the Dumas method on a Flash 2000 NC Analyzer (Thermo Fisher Scientific, Waltham, MA, USA) in duplicate. In this study the N content is presented, rather than protein content, since the conversion factor (N content to protein content) was not known for the materials used.

### 2.5. Yield Calculations

Process yield of total N and of total dry and wet matter, for each specific process step and for the full process, was calculated as:

(1) $${\mathrm{Yield}}_{X,F}=\frac{X_{F,\mathrm{out}}}{X_{F,\mathrm{in}}}$$

where X is total N, total dry matter, or total wet matter, and F is the process step, or steps, for which the yield is calculated. The yields were calculated for each process replicate separately and the mean values ± standard deviation are presented, with the numbers of replicates as stated in the method descriptions above.

### 2.6. Calculation of Theoretical pI

The theoretical isoelectric point (pI) for spinach RuBisCO was determined using the online pI calculation tool ProtParam on the Expasy server (web.expasy.org/cgi-bin/protparam/protparam, accession data 5 June 2020) [34]. The amino acid sequences were taken from the UniProt database under the entries P00870 (small subunit) and P00875 (large subunit) (www.uniprot.org, accession date 5 June 2020).

### 2.7. Statistical Evaluation

Flows of N, dry matter, and total mass through the process for the different crops were compared using general linear model analysis of variance (ANOVA) with Kenward-Roger’s method and a significance level of *p* < 0.05, followed by Tukey post-hoc test. All statistical analyses were performed in RStudio version 1.4.1106 [35], using the function packages lme4, emmeans, lmerTest, multcomp, and multcompView. The Kenward-Roger´s method was chosen due to the complex correlation structures of the data [36].

## 3. Results and Discussion

### 3.1. Effect of Biomass Source on Protein Fractionation

**Figure 3:**
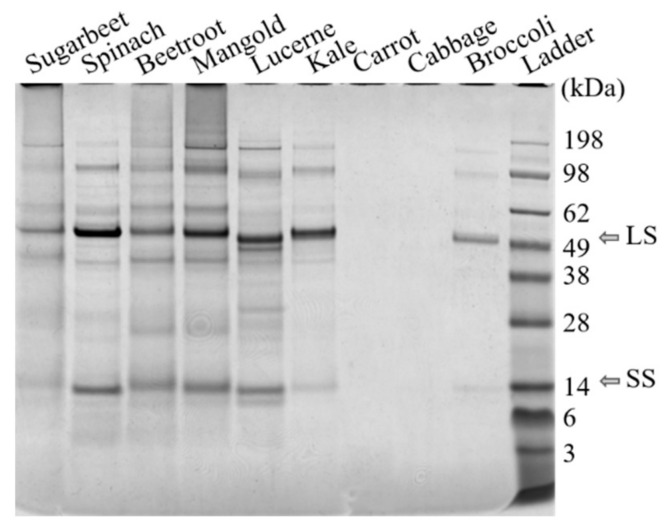
Results of SDS-PAGE analysis of freeze-dried white protein isolates (S4, see Figure 2) dissolved in water. The RuBisCO subunits are at ~55 kDa and ~14 kDa. LS: large subunit, SS: small subunit.

Protein fractionation with the selected method was successful for seven out of nine of the green biomass sources evaluated, as demonstrated by the N yield from the original biomass (BM) to re-dissolved white protein (S4) (Table 2a, Supplementary Figure S2). For carrot and cabbage protein fractionation was not successful, as shown by the low N yield and lack of RuBisCO in S4 from these crops (Figure 3). Thus, the origin of green leafy biomass had a large impact on the protein fractionation outcome, contradicting the hypothesis that any green leafy biomass can be used as raw material in a general protein extraction process. Other studies have achieved successful protein extraction from, e.g., cabbage, by applying different extraction methods [33].

**Figure 4:**
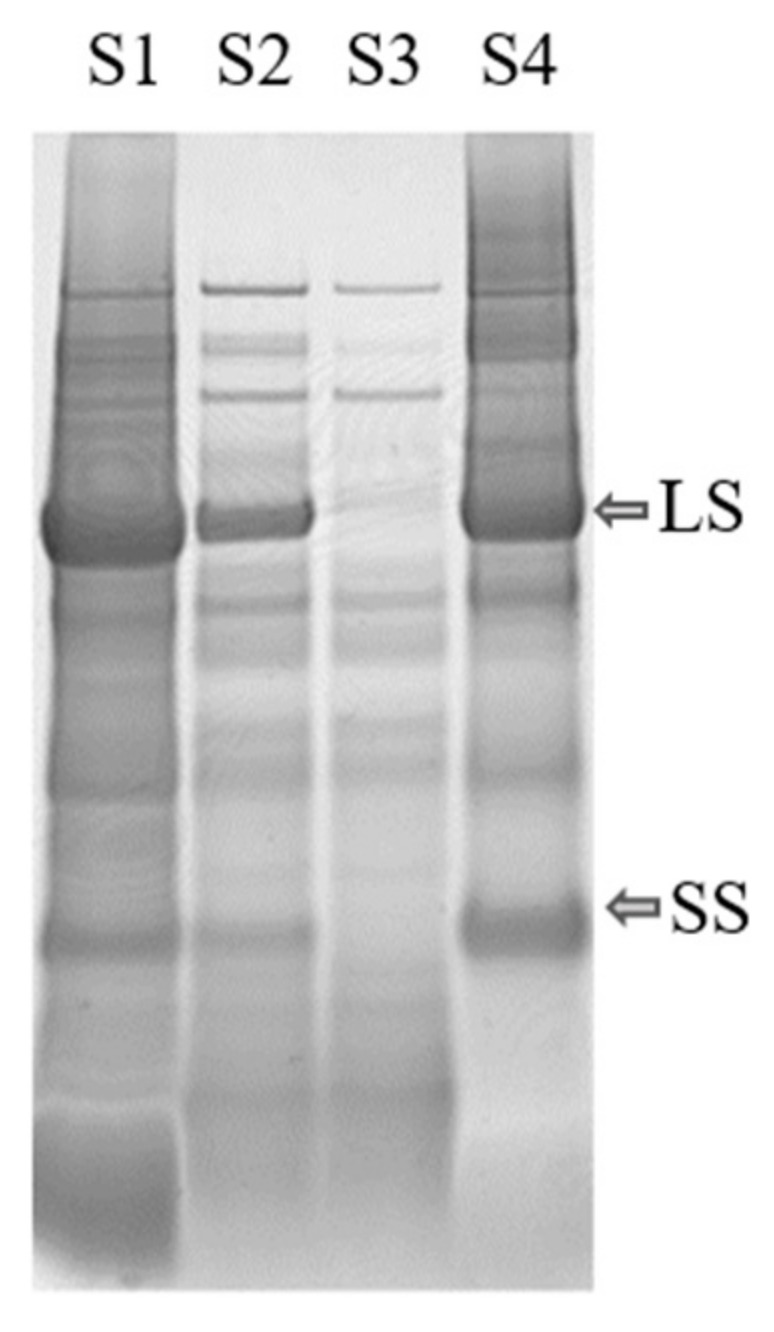
Protein content in mangold leaves, as determined by SDS-PAGE analysis of supernatants S1–S4 from the extraction process (see Figure 2). LS: large subunit, SS: small subunit of RuBisCO.

The protein fractionation results also differed significantly for the biomass types that were successfully fractionated (Table 2a), confirming that biomass source had a large impact on the outcome of the fractionation process. Presence of RuBisCO was detected in all biomass types, in the white juices (Figure 4, Supplementary Figure S1), and in the white protein fraction of the substrates for which the process was successful (Figure 3).

Of the nine biomass types evaluated, mangold leaves resulted in the highest N yield after the full process, with 1.9% of initial N in the biomass recovered in the white extract (S4) (Table 2a). The corresponding value for lucerne, the crop with the second highest level, was 1.5%. The highest yield of soluble N from the particle-free green juice (S1) to the final white protein (S4) (Supplementary Table S1) was seen for sugarbeet (7.5%). Previous studies evaluating white protein extracts from green biomass often report higher yields than those obtained in the present study. However, comparison of N yields between studies was hampered by the fact that (i) extraction methods generally differ between studies, (ii) a limited number of crop biomass types (mainly sugarbeet, lucerne, and spinach) have been extensively studied in this regard, and (iii) different combinations of methods and fractions are used for the calculations. Thus, results are rarely comparable in practice. In a previous study, juicing of sugarbeet leaves followed by acid and heat precipitation of the white protein fraction (a procedure partly comparable to steps 1 and 5 in Figure 2) resulted in N yield of ~25% [16]. In another study using sugarbeet leaves, heat precipitation of the green protein fraction and ultracentrifugation to concentrate the white protein (steps 1 and 4 in Figure 2) resulted in N yield of ~12% [11]. Thus, the fraction used to determine the N yield in those two studies was the most similar to our S2 fraction (WJ), but not our final resolubilized extract S4 (Table 2a). The N yield in S2 in the present study was 24.6% for mangold, 20.0% for lucerne, and 11.7% for sugarbeet, which is comparable to previous findings [11,16].

Mangold and lucerne not only had the highest N yields in this study, but also the highest N content in the white protein isolate (S4), with values of 4.0% and 3.6%, respectively (Table 2c). Higher N content has been reported in other studies fractionating white protein from sugarbeet, e.g., 9.6% and 7.6% [16,28], and from lucerne, e.g., 11.2%, 10.7%, 11.8%, and 14.8% [11,15,24,37]. As noted above for N yield, comparisons between studies are difficult, although a product mostly corresponding to fraction P3 in this study is often used as an end-point in other studies. The N content in P3 was between 5.1 and 12.5% for the different crops evaluated, corresponding well with previous findings.

To understand and characterize the background and reasons for differences in protein fractionation between the nine green biomass types studied, in this study additional steps were included in the standard fractionation method (as outlined in Figure 2).

### 3.2. Differences in Protein Fractionation between Biomass Types

#### 3.2.1. Juice Pressing

Juice pressing resulted in large variation between the crops in terms of N, total mass, wet mass, dry matter, and N yield, etc., from the biomass to GJ (Table 2), even for crops of the same species, e.g., for the *Beta vulgaris* varieties the N yield ranged from 15% for sugarbeet to 53% for mangold (Table 2, Supplementary Table S1). Variation in N yield between the biomass types evaluated in this study may be partly explained by structural differences in the biomass of various crops in terms of cell wall strength and water content. Previous studies have shown a significant negative correlation between maturity and protein extractability in other crops, e.g., clover, timothy, and chicory [38], so differences in maturity and cell wall thickness might also partly explain the differences between crops in this study. Therefore, factors such as biomass structural features, crop maturity, and cell wall thickness should be considered in industrial applications involving green biomass protein fractionation, especially when using crops, such as lucerne, which may be harvested several times during the season.

Higher total N yields were achieved from all biomass types when an additional juicing step was included (i.e., adding water to the extruded pulp and re-feeding it to the screw press), although the recovery varied (Table 2a). The effect was most prominent for lucerne, where the second press resulted in an N yield of 15% from BM to GJ_sp_. This increased the total N yield from juicing of lucerne to 67% (GJ + GJ_sp_), which is similar to previous findings of 47% and 50% N yield in GJ for lucerne [24,25] and 69% for sugarbeet [18]. A second press caused further cell disruption, which released soluble proteins, and the added water carried the protein into the GJ_sp_, which is critical when aiming for high N yield [25]. Overall, lucerne gave the highest N yield and carrot and sugarbeet the lowest after the two presses, with all cases showing substantially increased total N extraction from BM after the second pressing. Thus, a second juice pressing was beneficial for all biomass types evaluated, increasing the efficiency of the protein fractionation process.

Solid particles in the GJ were removed through centrifugation, resulting in a substantially particle-free green juice (S1) that was still green in color for all biomass types. This step was added to examine how water-soluble white proteins were separated in the following thermal precipitation step, in which any particles present in the GJ would be separated together with the green protein fraction. The centrifugation step was identified as the main reason for the unsuccessful protein fractionation of carrot and cabbage, resulting in low white protein yield. Less than 30% of the protein content in GJ was found in S1 after the centrifugation step for these two crops. For all other crops the transfer rate from GJ to S1 exceeded 50%, which corresponds to results reported for lucerne in a previous study [25]. The highest GJ to S1 recovery rates were found for spinach, lucerne (both ~80%), and sugarbeet (>90%). The low rates found for cabbage and carrot indicated poor cell breakage, high content of proteins bound to, or contained in, solid particles/organelles, or high content of insoluble proteins. This issue needs to be resolved for crops such as cabbage and carrot before they are suitable green biomass substrates for protein fractionation.

#### 3.2.2. Green Protein Fractionation and White Juice Production

**Figure 5:**
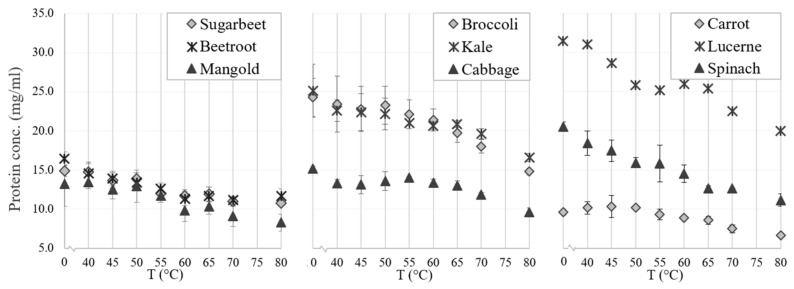
Effects of thermal treatment temperature on protein concentration (analyzed with the bicinchoninic acid method) in particle-free supernatant (fraction S1) from crop biomass.

**Figure 6:**
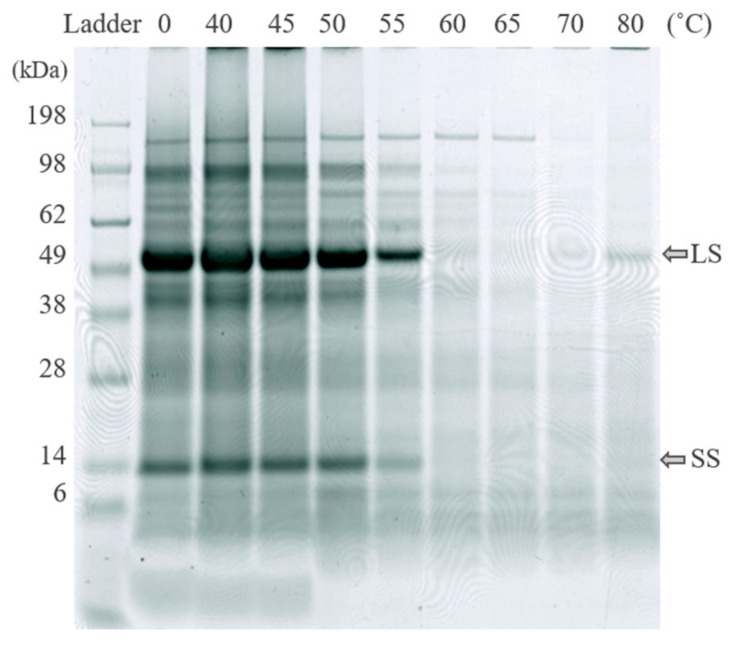
Effects of thermal treatment temperature on protein composition (analyzed using SDS-PAGE) in particle-free supernatant (fraction S1) from sugarbeet biomass. LS: large subunit, SS: small subunit of RuBisCO.

There were differences in thermal coagulation behavior of GJ from the different green biomass types, as indicated by the optimal temperature for precipitating the green proteins while simultaneously keeping the white protein in the WJ (Figure 5). The RuBisCO subunits (around 55 kDa and 14 kDa) and most other proteins in all biomass types were generally unaffected by heating for 25 min at temperatures of up to 50 °C, while protein bands started to disappear (precipitate together with the green proteins) at 50–55 °C and disappeared (precipitated fully) at 60–65 °C (Figure 5 and Figure 6). The reason for the weak reappearance of the RuBisCO large subunit at 80 °C for some biomass types (e.g., broccoli and sugarbeet; Figure 6) is unclear but could possibly be a result of the two subunits dissociating at higher temperature, and a difference in the solubility of the two subunits at these temperatures. The clearest difference between the biomass types with regard to precipitation temperature of RuBisCO was the upper temperature for full precipitation. RuBisCO was still present to some extent at 55 °C in all biomass types evaluated (Figure 5, Supplementary Figure S1), but at 65 °C RuBisCO was still only clearly present in the spinach sample. Similarities in response to thermal treatment (e.g., changes in protein composition and concentration) were seen for crops of the same family, e.g., the three varieties of *Beta vulgaris* (sugarbeet, beetroot, and mangold) and the two successfully extracted *Brassica* species (broccoli and kale). The *Beta vulgaris* varieties showed relatively low protein thermal stability, with a clear drop in protein concentration at 55 °C (Figure 5) and a noticeable decrease in the intensity of the RuBisCO bands at temperatures above 50 °C (Supplementary Figure S1). A similar concentration drop at 55 °C was observed for kale and broccoli (Figure 5), but the band intensity was intact at temperatures up to 55 °C (Supplementary Figure S1), indicating greater thermal stability of RuBisCO in *Brassica* biomass. Previous studies have determined the denaturation temperature of RuBisCO from lucerne to be between 61.85 °C and 66.85 °C, depending on the environment [39], and that of RuBisCO from spinach to be 64.9 °C [20]. Thus, differences in denaturation and precipitation of RuBisCO in the biomass types examined in this study might be explained by the environment of the protein, with the S1 matrix affecting the stability and thermosensitivity of RuBisCO and other proteins present. For successful thermal protein precipitation, the optimal temperature may have to be determined for each biomass type to be used in an industrial process.

Effects of duration of thermal treatment on protein precipitation from S1 of the different biomass types were not assessed in detail, but initial experiments indicated that thermal treatment duration may be important (results not shown). Previous studies have reported that lucerne GJ treated at 50 °C for 5 min is still green, but after 20 min the supernatant is clear [26], and that heating sugarbeet GJ at 55 °C for 5 min gives higher white protein yield than heating for 10 min [28]. Further studies are required to identify the optimal time and temperature settings for each of the nine biomass types evaluated here.

The N yield obtained from thermal precipitation (relative amount of N in S1 transferred to fractions S2 and P2) differed significantly between the biomass types. The N yield from S1 to S2 exceeded 80% for all biomass types except lucerne, mangold, and sugarbeet, for which the yield was considerably lower (52–61%) (Table 2b). These differences likely reflect variation in thermal sensitivity of the multiple proteins in S1 of the different biomass types.

#### 3.2.3. Acid Precipitation of the White Protein Fraction

The N yield from acid precipitation at pH 4.5 (S2 to P3) varied between 10.5% and 21.5% for most biomass types, but the values for cabbage and carrot were considerably lower (1.2% and 2.8%, respectively) (Table 2b). The N content of the acid-precipitated protein in the resulting pellet (P3) was around 8% for all crops except cabbage, carrot, and sugarbeet, for which the value was between 1.1% and 2.6% (Table 2c). Low N yield and N content of cabbage and carrot after precipitation was expected, based on the low yields already observed during particle separation of GJ to obtain S1 (i.e., low BM to S1 value; Table 2a). For the other biomass types, variations in precipitated protein amount may be the result of differences in the most suitable pH for precipitation. Previous studies have reported pH 4 or lower as optimal for precipitation of proteins in sugarbeet [23,24] and pH 3.5 as optimal for lucerne proteins [37].

**Figure 7:**
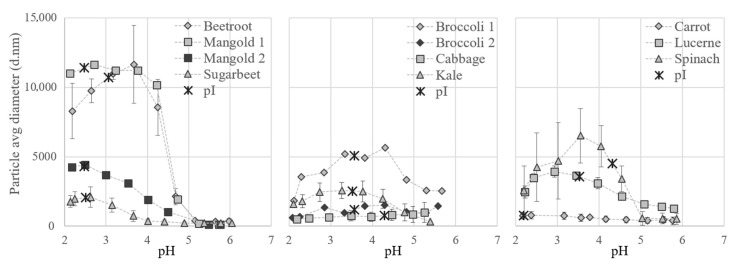
Particle size of white protein in thermally treated supernatant (fraction S2) from the different crops at different pH values during titration. Isoelectric point (pI) of S2 is marked on the respective curve.

Particle size measurements and pI determinations confirmed that proteins from the different biomass types responded differently to variations in pH (Figure 7). Differences were even seen between varieties, e.g., the particle size of beetroot aggregates increased drastically at pH 4.5 and those of sugarbeet at pH 3.5. Data on pI values and on solubility of the full S2 fraction have not been reported previously, but the pH for minimum solubility of the final white protein concentrates has been reported to be e.g., 3.5 for soy bean leaves [40], 4 for spinach [31], and 5 for sugarbeet [11].

For all crops evaluated except cabbage, protein aggregates were formed during titration with acid and the particles generally increased in size at pH values just below 5 and decreased in size at values below 2.5 (Figure 7). Precipitation, causing the formation of particles, occurs when the charge on amino acids reaches a net zero state; the isoelectric point (pI) [41]. The pI for the S2 fractions from the different crops ranged between 2.2 and 4.3, according to the zeta potential measurements (Figure 7). The pI for spinach S2 was 4.3, which is considerably lower than the theoretical pI value for pure spinach RuBisCO of 6.03. This discrepancy is most likely related to the presence of other proteins and compounds in the solution, which would have a large effect on the net charge.

The minimum solubility, and also the largest aggregates of the proteins, were expected around the pI, but for several of the crops the particle size increased before pI was reached during titration. This indicates that some proteins present, e.g., RuBisCO, started to precipitate earlier in the titration, i.e., at higher pH. For example, precipitation of RuBisCO from mangold clearly occurred at pH 4.5 (see Figure 4), since RuBisCO was not present in S3 but was present in S2, even though the maximum particle size (measured by dynamic light scattering) occurred close to pH 2.5 (Figure 7), which was the pI of the overall solution.

To achieve sufficient separation of white proteins, the particle size did not necessarily need to reach the maximum. For all biomass types studied here, aggregation was initiated at pH 4.5 (the pH used for precipitation of the white proteins). When acid precipitation is chosen as a method for separating white proteins in a biorefinery set-up, the same pH could be used for different biomass types, but the yields would most likely benefit from individual adjustments.

#### 3.2.4. Resolubilization of the White Protein Fraction

The N content in the resulting resolubilized, neutralized, and freeze-dried white protein concentrate (S4) varied for the different biomass types evaluated. It was highest for lucerne (4%) and lowest for carrot and cabbage (0.6% and 0.9%, respectively) (Table 2c). Again, the low levels for carrot and cabbage were the result of incomplete protein fractionation in earlier steps of the process. The other biomass types resulted in white protein concentrates with N content ranging from 2.3% to 3.6%. Higher N content could be achieved by adding a washing step for the acid-precipitated protein (P3) prior to resolubilization, as this would remove some of the co-precipitated compounds present. Several factors are known to have an impact on resolubilization of the white protein fraction, including pH, treatment temperature, use of enzymes, enzyme inhibitors, and adsorptive resins that influence the impact of phytochemicals on protein resolubilization [22,40,42,43]. The variation in protein resolubilization of the P3 fraction between biomass types might thus be the result of differences in phytochemicals binding to the proteins. Use of enzymes or enzyme inhibitors might result in more similar white protein yield for different biomass types.

### 3.3. Use of Green Biomass in Industrial Protein Fractionation for Food Ingredients, Additives, and Products

The long-term goal behind the present study was a desire to set up an industrial process for protein fractionation of green biomass that could contribute high-value food ingredients, additives and products. Such a process would require green biomass, from a range of different sources, to be available throughout the year. Additionally, such an upscaling would require a protocol suitable for a large range of green biomass types, to allow for maximum facility utilization throughout the year. Therefore, a general protocol based on literature methods [18,24,28,31] was used in this study. However, the present study clearly revealed differences in protein fractionation between green biomass of different origin, suggesting a need for a crop-specific (and eventually also growth stage-specific) fractionation procedure in order to achieve successful production of high-value proteins suitable for food. This study identified important steps to consider for industrial protein fractionation of each of the biomass types, although individual biomass-based settings in an industrial production plant may result in higher production costs. A year-round availability of green biomass, of similar growth stage, would also require large storage facilities for e.g., frozen [44] or silage green biomass. Such a storage will also highly affect the costs (storage of frozen biomass) or destroy the proteins (silage).

Analysis of the protein composition by SDS-PAGE (Figure 3 and Figure 4) showed a high content of RuBisCO in the initial particle-free green juice (S1), the thermally treated S1 (S2), and the final resolubilized protein (S4), while the supernatant remaining after acid precipitation (S3) contained negligible amounts, indicating successful RuBisCO separation. From a food perspective, the RuBisCO-rich white protein concentrate is highly interesting, as it is known to be highly nutritious and have promising functional food properties [11,12,13]. However, the green protein fraction (P1 combined with P2) and other fractions obtained from the protein fractionation might also be relevant co-products, not least for further fractionation of interesting compounds. Phenolic compounds were present to various degrees in all fractions (data not shown), and these might be of interest as antioxidant-enriched products or for further fractionation before use in food and biomedical applications [45,46]. Fractionation of additional high-value compounds from the green biomass may contribute positively to the economy for the whole process [46].

## 4. Conclusions

Green biomass source substantially influenced the outcome, when a general protein fractionation procedure was applied to extract high-quality protein for use by the food industry. White protein concentrate rich in RuBisCO was extractable from a majority of nine green leafy biomass types subjected to general fractionation, although with considerable variation in protein yield and quality. Biomass type affected protein yield all fractionation steps, i.e., juicing, thermal precipitation, acid precipitation, and resolubilization. Factors such as biomass structural features, crop maturity, and cell wall thickness probably affected the outcome of the juicing step, with stronger structures needing harsher treatment. Protein from the different biomass sources associated to particles in the green juice to varying degrees, with a strong association of proteins for carrot and cabbage leaves, resulting in low yield of high-quality food protein. Modification of the protein fractionation procedure is required to release such protein in these biomass types.

The biomass matrix influenced the thermosensitivity of RuBisCO and other proteins present in the biomass and was important for the thermal precipitation step. Similarities in thermosensitivity were seen for biomass of related origin, e.g., proteins from different varieties of *Beta vulgaris* were generally more sensitive to heat than proteins from, e.g., lucerne. For biomass of sugarbeet, lucerne, kale, and broccoli, pH < 4.5 was most suitable for acid precipitation, while for mangold, beetroot and spinach pH of 4.5 was most suitable. This indicates that differences in the protein environment influenced acid precipitation behavior, as RuBiSCO theoretically precipitates at around pH 6.

In a biorefinery context, use of a general procedure, but with some parameters modified for each new biomass type, would improve the final outcome compared with using a general procedure for all green leafy biomass.

## Figures and Tables

**Table 1:** The nine types of agricultural green biomass evaluated in this study and their nitrogen (N) content on a dry matter (DM) basis.

Biomass Source		Collection Date	%N	%DM
Broccoli *	*Brassica oleracea,* var. *italica*	2 October 2017	3.2	13.3
Cabbage *	*Brassica oleracea,* var. *capitata*	30 August 2017	2.1	11.0
Kale *	*Brassica oleracea,* var. *sabellica*	23 October 2017	3.0	13.3
Mangold	*Beta vulgaris,* subsp. *vulgaris,* var. *cicla*	30 August 2017	2.1	8.4
Beetroot *	*Beta vulgaris,* subsp. *vulgaris,* var. *Red hawk*	13 September 2017	3.2	9.9
Sugarbeet *	*Beta vulgaris,* subsp. *vulgaris,* var. *Lombok*	12 October 2018	3.0	13.0
Carrot *	*Daucus carota* subsp. *sativus*	28 June 2018	2.1	17.7
Lucerne	*Medicago sativa*	25 May 2018	2.8	20.9
Spinach	*Spinacia oleracea*	Retail **	4.8	10.6

* True harvest residues; ** Italian produce.

**Table 2:** (**a**) Yield (%) of nitrogen (N) relative to N content in initial biomass (BM) in the different process steps (see Figure 2). Mean of three process replicates (unless otherwise indicated by superscript numbers). (**b**) Yield (%) of N relative to the ingoing material for each process step. (**c**) N content (%) on a dry matter basis (average of replicates) of the flows in the extraction process. Different letters (A–D) indicate significant differences (*p* < 0.05) in yield. Superscript numbers indicate numbers of replicates if not triplicates. GJ: green juice, GJ_sp_: green juice from the second press, S1: particle-free green juice, P_fp_: pellet with freeze-thaw precipitate, S2: supernatant after thermal treatment, P3: acid-precipitated white protein, S4: resolubilized protein fraction, P4: insoluble fraction.

(a)	Process Step										
Yield of N (%)	BM Pressing		Second BM Pressing		Separation of Particles	Thermal Precipitation	Acid Precipitation		Full Process		
Biomass	BM to GJ		BM to GJ_sp_		BM to S1	BM to S2	BM to P3		BM to S4		
Broccoli	28.2 ± 2.0 ^BC^		7.2± 1.5 ^BC^		14.8 ± 1.4 ^BC^	15.5 ± 2.2 ^B^	2.9 ± 0.7 ^CD^		0.4 ± 0.0 ^A^		
Cabbage	37.4 ± 5.4 ^C^		7.9 ± 2.0 ^BC^		9.3 ± 2.4 ^AB^	14.4 ± 3.1 ^AB^	0.2 ± 0.1 ^AB^		0.2 ± 0.1 ^A^		
Kale	30.3 ± 3.3 ^BC^		5.0 ± 0.2 ^AB^		19.3 ± 0.7 ^CD^	18.7 ± 0.7 ^BC^	2.1 ± 0.2 ^ABCD^		0.5 ± 0.1 ^AB^		
Mangold	53.1 ± 1.0 ^D^		8.4 ± 0.6 ^C^		36.9 ± 1.3 ^E^	24.6 ± 4.6 ^C^	3.7 ± 1.6 ^D^		1.9 ± 1.1 ^B^		
Beetroot	35.5 ± 2.1 ^C^		6.3 ± 0.4 ^ABC^		21.9 ± 2.1 ^D^	17.5 ± 0.3 ^BC^	3.8 ± 0.1 ^D^		1.0 ± 0.5 ^AB^		
Sugarbeet	14.6 ± 4.0 ^A^		3.7 ± 0.6 ^A^		12.1 ± 1.5 ^AB^	11.72 ± 2.4 ^AB^	1.2^2^ ± 0.3 ^ABC^		0.9^2^ ± 0 ^AB^		
Carrot	20.9 ± 2.7 ^AB^		5.3 ± 0.4 ^AB^		6.0 ± 0.5 ^A^	7.2 ± 0.4 ^A^	0.2 ± 0.2 ^A^		0.1 ± 0.1 ^A^		
Lucerne	52.1 ± 5.4 ^D^		15.1 ± 0.9 ^D^		42.9 ± 4.4 ^E^	20.0 ± 0.9 ^BC^	3.3^1 BCD^		1.5^1 AB^		
Spinach	27.6 ± 5.0 ^BC^		8.9 ± 1.3 ^C^		25.1 ± 0.4 ^D^	18.0 ± 3.8 ^BC^	3.0^2^ ± 0.1 ^CD^		1.0^2^ ± 0.4 ^AB^		
**(b)**	**Process Step**										
**Yield of N (%)**	**Separation of Particles**		**Separation of Freeze-Thaw Precipitate**			**Thermal Precipitation**			**Acid Precipitation**		
Biomass	GJ to S1		S1 to P_fp_			S1 to S2			S2 to P3		
Broccoli	52.6 ± 4.2 ^AB^		12.6 ± 1.9 ^A^			84.2 ± 0.5 ^CD^			18.8 ± 1.7 ^BC^		
Cabbage	24.9 ± 5.9 ^A^		42.2 ± 14.1 ^B^			89.9 ± 1.8 ^DE^			1.2 ± 1.1 ^A^		
Kale	64.1 ± 6.4 ^BC^		6.5 ± 1.4 ^A^			84.3 ± 2.5 ^CD^			11.4 ± 1.2 ^B^		
Mangold	69.6 ± 3.6 ^BC^		4.6 ± 3.9 ^A^			52.0 ± 6.0 ^A^			15.2 ± 5.4 ^BC^		
Beetroot	61.6 ± 3.5 ^BC^		6.0 ± 6.0 ^A^			84.9 ± 16.1 ^CD^			21.6 ± 0.3 ^C^		
Sugarbeet	79.7 ± 31.4 ^BC^		1.2 ± 0.2 ^A^			61.4^2^ ± 13.2 ^ABC^			10.5^2^ ± 0.5 ^AB^		
Carrot	29.2 ± 5.1 ^A^		1.8 ± 0.1 ^A^			110.6 ± 8.0 ^E^			2.8 ± 2.4 ^A^		
Lucerne	82.5 ± 8.5 ^BC^		7.7 ± 1.5 ^A^			52.2 ± 4.4 ^AB^			16.1^1 BC^		
Spinach	93.3 ± 17.9 ^C^		7.1 ± 0.7 ^A^			79.5 ± 10.1 ^BCD^			16.9^2^ ± 4.0 ^BC^		
**(c)**											
**% N**	**Flow in the Process**										
**Biomass**	BM *	**GJ**	**Pulp**	**GJ_sp_**	**Pulp_sp_**	**S1**	**P_fp_**	**S2**	**P3**	**S4**	**P4**
Broccoli	3.2	2.1 ± 0.1	1.9 ± 0.2	2.4 ± 0.1	1.9 ± 0.2	1.7 ± 0.1	5.8 ± 0.2	2.0 ± 0.2	7.9 ± 0.2	1.8 ± 0.4	13.6 ± 0.2
Cabbage	2.1	1.7 ± 0.2	1.2 ± 0.1	1.5 ± 0.3	1.3 ± 0.2	0.5 ± 0.1	8.2 ± 0.3	0.8 ± 0.2	1.2 ± 0.0	0.9 ± 0.1	0.7 ± 0.5
Kale	3	2.7 ± 0.2	1.5 ± 0.2	2.9 ± 0.1	2.2 ± 0.1	2.2 ± 0.0	1.7 ± 0.1	2.3 ± 0.1	7.9 ± 0.4	2.6 ± 0.4	12.3 ± 1.2
Mangold	2.1	2.3 ± 0.1	2.4 ± 0.4	2.6 ± 0.2	1.4 ± 0.1	1.9 ± 0.2	4.3 ± 0.3	1.6 ± 0.3	7.2 ± 0.6	3.6 ± 0.9	13.7 ± 0.4
Beetroot	3.2	3.0 ± 0.0	3.3 ± 0.2	3.6 ± 0.1	1.6 ± 0.2	2.6 ± 0.1	5.5 ± 0.3	2.2 ± 0.1	9.2 ± 0.7	3.3 ± 0.7	14.0 ± 0.3
Sugarbeet	3	1.1 ± 0.2	1.3 ± 0.3	1.6 ± 0.2	2.6 ± 0.5	1.0 ± 0.1	5.7 ± 0.4	1.1 ± 0.1	2.6^2^ ± 1.5	2.3^2^ ± 0.9	0.5^2^ ± 0.1
Carrot	2.5	1.5 ± 0.1	2.1 ± 0.0	2.3 ± 0.1	3.2 ± 0.3	0.6 ± 0.1	8.0 ± 0.2	0.6 ± 0.0	1.1 ± 0.4	0.6 ± 0.1	
Lucerne	2.8	3.8 ± 0.3	2.0 ± 0.1	4.1 ± 0.1	5.1 ± 0.2	4.3 ± 0.1	8.0 ± 0.0	3.2 ± 0.1	7.21	4.01	12.91
Spinach	5.1	4.4 ± 0.3	4.9 ± 0.2	4.1 ± 0.1	1.5 ± 0.3	4.2 ± 0.3	2.9 ± 0.5	4.0 ± 0.2	8.1 ± 1.8	3.3 ± 0.5	14.1 ± 0.5

* Biomass N composition was assumed to be the same in all process replicates. Standard deviations for biomass % N are found in Table 1.

## Data Availability

Not applicable.

## Supplementary Materials

The following are available online at https://www.mdpi.com/article/10.3390/foods10112533/s1, Figure S1: SDS-PAGE analyses of thermally treated particle free green juice (S2), Figure S2: Visualization of N yields in some of the process steps for the different biomass types, Table S1: Yields (in %) of total mass (m), dry matter (DM), and nitrogen (N), in the white protein extraction process.
