# Supplementary material for: Protein Fractionation of Green Leaves as an Underutilized Food Source—Protein Yield and the Effect of Process Parameters
Source: Foods. 2021 Oct 21;10(11):2533. doi: 10.3390/foods10112533 (PMC8622718; doi:10.3390/foods10112533)
Supplement: Supplementary file 1 [file foods-10-02533-s001.zip › foods-1406635-supplementary.pdf]

Supplementary Materials

For the paper: Protein fractionation of green leaves as an underutilized food source – protein yield and the effect of process parameters

By authors: Anna-Lovisa Nynäs, William R. Newson, Eva Johansson

Supplementary Figure S1. SDS-PAGE analysis of particle free green juice (S2) thermally treated at different temperatures.

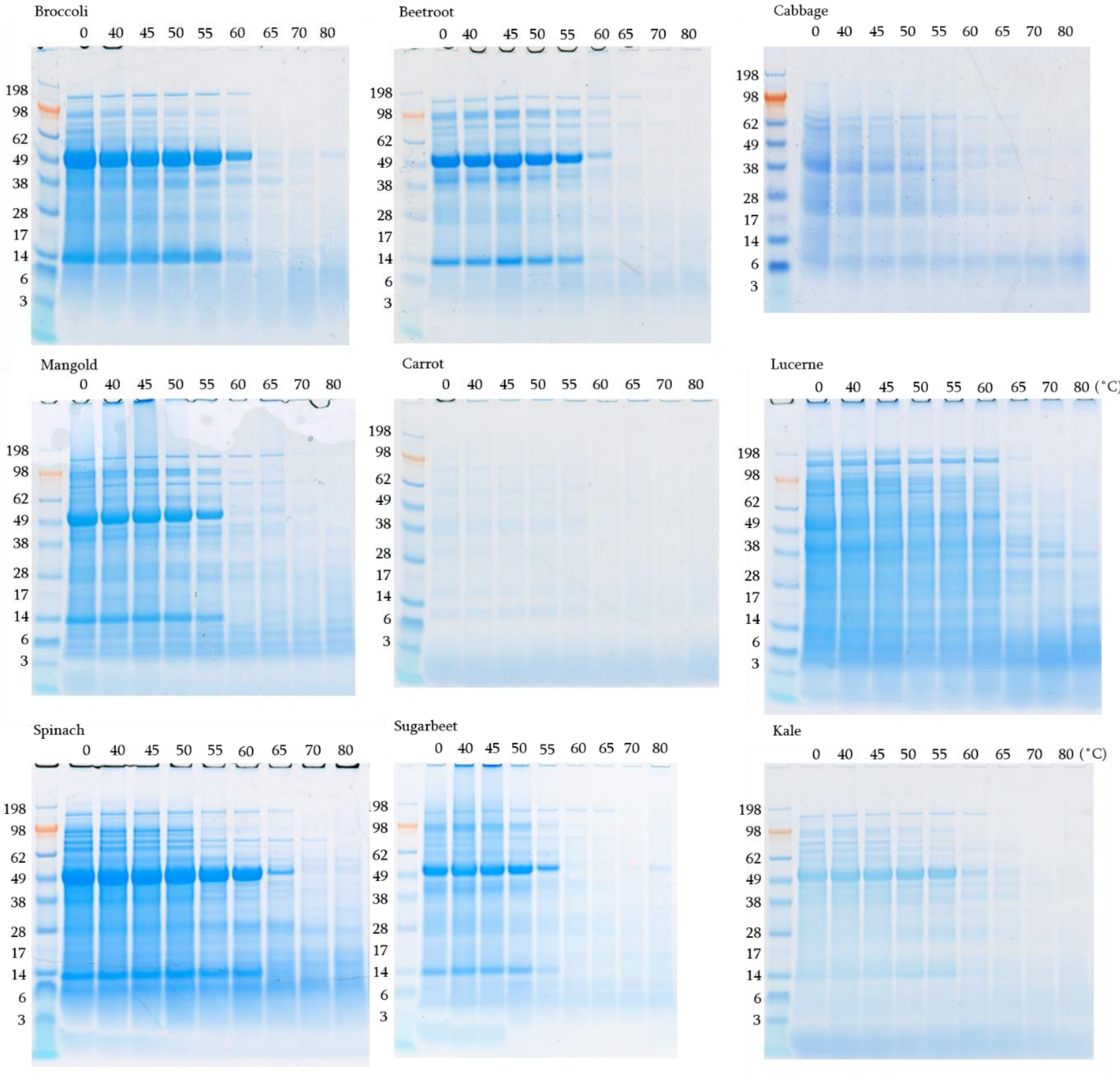

Supplementary Figure S2. Visualization of N yields in some of the process steps for the different biomass types.

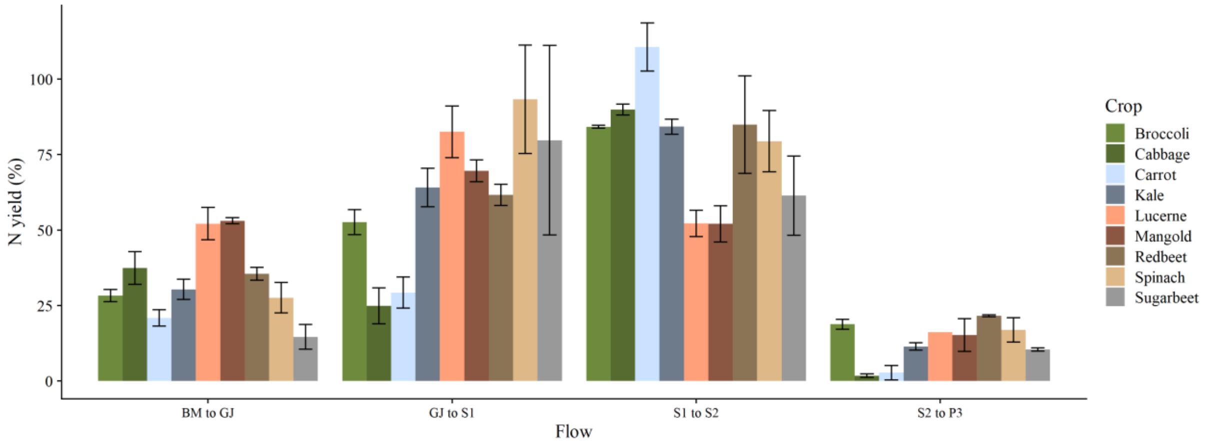

Supplementary Table S1. Yields (in %) of total mass (m), dry matter (DM), and nitrogen (N), in the white protein extraction process. The italic values are the standard deviations for the replicate treatments, and the superscripted values are the number of replicates if not three. BM: Biomass, GJ: Green juice, GJ_{sp}: GJ from second press, S1: particle free GJ, P_{fp}: Freeze-precipitated proteins from frozen and thawed S1, S2: Supernatant of thermally treated GJ, $P_{fp}2$: Freeze-precipitated proteins from frozen and thawed S2, P3: Acid precipitated white proteins, S4: Resolubilized and freeze-dried white proteins, tot. green frac.: Green particles and thermally precipitated green protein.

		Pressing of juice				Separation of green particles		Freeze precipitated protein		Thermal precipitation of green protein				Freeze precipitated protein	
		BM to GJ		BM to GJ_{sp}		GJ to S1		S1 to P_{fp}		GJ to S2		S1 to S2		S2 to $P_{fp}2$	
Broccoli	m	65.9	*1.7*	6.3	*0.9*	90.5	*1.2*			82.7	*2.5*	92.6	*1.7*		
	D	43.0	*2.2*	9.6	*1.7*	63.4	*3.1*	3.8	*0.8*	59.0	*2.3*	96.8	*0.9*	4.2	*0.8*
	N	28.2	*2.0*	7.2	*1.5*	52.6	*4.2*	12.6	*1.9*	54.8	*4.5*	84.2	*0.5*		
Cabbage	m	63.5	*2.4*	9.6	*1.9*	93.4	*0.7*			90.1	*2.3*	97.5	*1.8*		
	D	44.9	*1.3*	11.0	*0.5*	78.6	*3.6*	2.7	*0.1*	79.2	*3.9*	103.4	*1.7*	1.5	*0.4*
	N	37.4	*5.4*	7.9	*2.0*	24.9	*5.9*	42.2	*14.1*	38.4	*5.1*	89.9	*1.8*		
Kale	m	71.1	*0.3*	4.0	*0.5*	92.1	*0.3*			87.6	*0.7*	96.5	*1.2*		
	D	34.8	*1.6*	5.3	*0.2*	77.1	*3.9*	3.3	*0.5*	71.8	*2.2*	95.5	*2.7*	2.6	*0.3*
	N	30.3	*3.3*	5.0	*0.2*	64.1	*6.4*	6.5	*1.4*	62.0	*4.4*	84.3	*2.5*		
Mangol	m	72.5	*2.0*	5.4	*1.3*	95.0	*0.6*			88.2	*0.8*	93.3	*1.3*		
	D	50.0	*3.2*	6.9	*0.8*	84.0	*2.2*	1.5	*1.2*	67.7	*5.8*	81.8	*5.4*	1.4	*0.8*
	N	53.1	*1.0*	8.4	*0.6*	69.6	*3.6*	4.6	*3.9*	46.4	*9.5*	52.0	*6.0*		
Redbeet	m	74.1	*1.2*	5.1	*0.7*	95.1	*0.4*			89.8	*1.6*	95.7	*1.5*		
	D	37.6	*2.4*	5.6	*0.3*	71.9	*6.2*	2.0	*1.9*	68.1	*2.7*	99.1	*7.3*	1.0	*0.6*
	N	35.5	*2.1*	6.3	*0.4*	61.6	*3.5*	6.0	*6.0*	49.4	*3.0*	84.9	*16.1*		
Sugarbee	m	69.5	*1.3*	6.7	*0.6*	95.5	*0.6*			90.0	*1.9*	95.0	*2.5*		
	D	39.7	*5.6*	7.2	*0.5*	93.9	*9.3*	0.6	*0.3*	83.6	*13.0*	89.6	*10.5*	2.5	*1.8*
	N	14.6	*4.0*	3.7[2]	*0.6*	79.7[2]	*31.4*	1.2[2]	*0.2*	73.5	*5.4*	61.4[2]	*13.2*		
Carrot	m	67.7	*0.7*	3.4	*0.8*	87.5	*0.1*			86.0	*1.3*	98.6	*1.4*		
	D	36.1	*2.2*	5.8	*0.6*	73.9	*3.5*	0.6	*0.0*	78.3	*4.0*	106.6	*0.6*	1.0	*0.2*
	N	20.9	*2.7*	5.3	*0.4*	29.2	*5.1*	1.8	*0.1*	35.0	*5.3*	110.6	*8.0*		
Lucerne	m	62.8	*1.6*	5.6	*0.5*	88.5	*1.3*			64.8	*0.5*	76.6[2]	*0.2*		
	D	37.3	*1.1*	10.2	*0.6*	74.1	*6.3*	5.9	*0.9*	46.8	*0.5*	70.0[2]	*5.2*	5.7[1]	-
	N	52.1	*5.4*	15.1	*0.9*	82.5	*8.5*	7.7	*1.5*	40.0	*3.0*	52.2[2]	*4.4*		
Spinach	m	54.9	*8.4*	8.7	*5.4*	94.6	*1.0*			83.2	*3.8*	90.2[2]	*3.4*		
	D	31.5	*4.3*	10.9	*1.4*	98.0	*21.6*	3.9	*0.5*	67.0	*9.6*	81.3[2]	*9.0*	0.3[1]	-
	N	27.6	*5.0*	8.9	*1.3*	93.3	*17.9*	7.1	*0.7*	59.3	*15.0*	79.5[2]	*10.1*		

		Acid precipitation of white protein				Yield of white protein						Yield of green protein			
		S2 to P3		BM to P3		S1 to S4		S2 to S4		BM to S4		BM to tot. green frac.		GJ to tot green frac.	
Broccoli	m	2.1	*0.4*	1.2	*0.2*							7.0	*0.6*	10.7	*1.2*
	D	4.6	*0.8*	1.2	*0.3*	2.9	*0.7*	3.1	*0.7*	0.8	*0.2*	14.0	*1.2*	32.5	*1.0*
	N	18.8	*1.7*	2.9	*0.7*	2.9	*0.2*	2.8	*0.1*	0.4	*0.0*	13.1	*1.7*	46.4	*3.6*
Cabbage	m	1.2	*0.2*	0.7	*0.1*							4.4	*0.3*	6.9	*0.7*
	D	1.5	*0.5*	0.5	*0.1*	1.7	*0.6*	1.7	*0.6*	0.6	*0.2*	9.6	*1.0*	21.3	*1.5*
	N	1.2	*1.1*	0.2	*0.1*	2.8	*1.3*	1.6	*0.2*	0.2	*0.1*	16.5	*0.7*	44.5	*5.7*
Kale	m	1.6	*0.1*	1.0	*0.0*							6.0	*0.3*	8.5	*0.5*
	D	3.3	*0.4*	0.8	*0.1*	2.3	*0.5*	2.5	*0.6*	0.6	*0.2*	8.1	*0.2*	23.4	*0.4*
	N	11.4	*1.2*	2.1	*0.2*	2.6	*0.4*	2.7	*0.5*	0.5	*0.1*	12.9	*0.7*	42.6	*2.5*
Mangol	m	1.8	*0.8*	1.1	*0.6*							4.3	*0.4*	6.0	*0.6*
	D	3.3	*1.5*	1.1	*0.5*	2.6	*1.2*	3.2	*1.3*	1.1	*0.4*	10.1	*1.0*	20.1	*0.9*
	N	15.2	*5.4*	3.7	*1.6*	5.1	*2.8*	7.5	*3.3*	1.9	*1.1*	23.5	*0.8*	44.2	*0.7*
Redbeet	m	2.4	*0.5*	1.6	*0.3*							4.2	*0.4*	5.6	*0.5*
	D	5.2	*0.6*	1.3	*0.1*	3.7	*1.5*	3.8	*1.3*	1.0	*0.3*	9.0	*1.2*	23.7	*1.7*
	N	21.6	*0.3*	3.8	*0.1*	4.9	*2.7*	6.0	*2.9*	1.0	*0.5*	14.3	*1.9*	40.0	*3.0*
Sugarbee	m	1.7	*0.7*	1.1	*0.4*							3.7	*0.3*	5.4	*0.4*
	D	4.1	*1.7*	1.3	*0.4*	2.9	*0.8*	3.3	*1.4*	1.1	*0.3*	6.7	*0.6*	17.0	*1.2*
	N	10.5[2]	*0.5*	1.2[2]	*0.3*	7.5[2]	*1.3*	7.8[2]	*1.3*	0.9[2]	*0.0*	7.1[2]	*1.4*	49.6[2]	*5.2*
Carrot	m	1.0	*0.1*	0.6	*0.1*							8.3	*0.0*	12.3	*0.2*
	D	1.4	*0.8*	0.4	*0.2*	1.4	*0.8*	1.3	*0.8*	0.4	*0.2*	11.9	*0.3*	33.0	*1.1*
	N	2.8	*2.4*	0.2	*0.2*	1.6	*0.8*	1.3	*0.7*	0.1	*0.1*	22.8	*0.7*	110.3	*13.2*
Lucerne	m	3.8[1]	-	1.5[1]	-							11.0[2]	*0.3*	17.7[2]	*0.7*
	D	7.0[1]	-	1.2[1]	-	3.9[1]	-	5.6[1]	-	1.0[1]	-	14.1[2]	*0.7*	38.1[2]	*0.6*
	N	16.1[1]	-	3.3[1]	-	3.7[1]	-	7.2[1]	-	1.5[1]	-	17.6[2]	*1.0*	35.1[2]	*2.1*
Spinach	m	3.6[2]	*1.0*	1.5	*0.2*							4.1[2]	*0.2*	8.2	*1.4*
	D	8.5[2]	*3.6*	1.9[2]	*0.5*	5.8[2]	*3.7*	7.8[2]	*5.6*	1.7[2]	*1.0*	6.2[2]	*0.6*	18.3	*2.2*
	N	16.9[2]	*4.0*	3.0[2]	*0.1*	4.2[2]	*1.8*	6.2[2]	*3.8*	1.0[2]	*0.4*	6.8[2]	*0.8*	22.4	*1.7*
